# LncRNA nuclear receptor subfamily 2 group F member 1 antisense RNA 1 (NR2F1-AS1) aggravates nucleus pulposus cell apoptosis and extracellular matrix degradation

**DOI:** 10.1080/21655979.2021.2016087

**Published:** 2022-01-30

**Authors:** Longlong Du, Xuefeng Li, Qimeng Gao, Puwei Yuan, Yindi Sun, Yingpu Chen, Bo Huang, Yu Deng, Baohui Wang

**Affiliations:** a Pain Area of Orthopedics of Traditional Chinese Medicine, Honghui Hospital, Xi’an Jiaotong University, Xi’an, Shaanxi, China; b Department of Traditional Chinese and Western Medicine, Shaanxi University of Traditional Chinese Medicine, Xianyang, Shaanxi, China; c Department of Orthopaedic, Affiliated Hospital of Jiujiang University, Jiujiang, Jiangxi, China; d Department of Orthopaedic Trauma and Microsurgery, Zhongnan Hospital of Wuhan University, Wuhan, Hubei, China

**Keywords:** Intervertebral disc degeneration, NR2F1-AS1, miR-145-5p, foxo1

## Abstract

Emerging reports uncover that long noncoding RNAs (lncRNAs) help regulate intervertebral disc degeneration (IVDD). Here, we probe the function of lncRNA nuclear receptor subfamily 2 group F member 1 antisense RNA 1 (NR2F1-AS1) in IVDD. Quantitative reverse transcription-polymerase chain reaction (qRT-PCR) was applied to verify the expression of NR2F1-AS1 and miR-145-5p in nucleus pulposus (NP) tissues from IVDD patients or NP cells dealt with IL-1β or TNF-α. Flow cytometry or the TdT-mediated dUTP nick end labeling (TUNEL) assay was performed to validate the apoptosis of NP cells with selective regulation of NR2F1-AS1 and miR-145-5p. ECM-related genes, FOXO1, Bax, and Bcl2 were evaluated by qRT-PCR or Western blot (WB). The targeted relationships between NR2F1-AS1 and miR-145-5p, miR-145-5p and FOXO1 were testified by the dual-luciferase reporter assay and the RNA immunoprecipitation (RIP) assay. Our outcomes substantiated that NR2F1-AS1 was up-regulated, while miR-145-5p was down-regulated in intervertebral disc tissues of IVDD patients or NP cells treated with IL-1β or TNF-α. Besides, overexpressing NR2F1-AS1 intensified ECM degradation and NP cell apoptosis induced by IL-1β, while knocking down NR2F1-AS1 or up-regulating miR-145-5p reversed IL-1β-mediated effects in NP cells. Meanwhile, NR2F1-AS1 choked miR-145-5p and abated its effects in NP cells. This study confirms that NR2F1-AS1 modulates IVDD progression by up-regulating the FOXO1 pathway through the sponge of miR-145-5p as a competitive endogenous RNA (ceRNA).

## 1. Introduction

Intervertebral disc degeneration (IVDD) is caused by repetitive mechanical loads or wear, leading to backache[1]. IVDD is characterized by increased levels of the proinflammatory cytokines secreted by intervertebral disc cells[2]. Current treatments for IVDD and backache include analgesics or surgery, which aim to reduce symptoms rather than eradicate the latent pathology [3]. However, the progression mechanism of IVDD has not been well clarified [4]. Recent reports have testified that long noncoding RNAs (lncRNAs) are powerful regulators of gene expression in IVDD [5]. Hence, studying the molecular mechanism of IVDD is expected to provide a novel option for its treatment.

LncRNAs have become vital modulators of multiple biological processes, including cell proliferation, apoptosis, inflammation, metabolic modulation, et al [6]. LncRNAs are abnormally expressed in tumors[7], inflammation[8], and cardiovascular diseases [9] and exert a unique role in different diseases by regulating various downstream targets (such as chromatin, RNA, and proteins). The latest research indicated that lncRNAs are implicated in the pathological processes of various bone diseases, including extracellular matrix (ECM) degradation, inflammation, apoptosis, and angiogenesis [10–12]. Thus, lncRNAs contribute to IVDD. As a vital member of lncRNAs, lncRNA nuclear receptor subfamily 2 group F member 1 antisense RNA 1 (NR2F1-AS1) is located at 5q15, with a length of 2814 bp. It is reported that NR2F1-AS1 affects tumor progression as an oncogene [13–15]. However, the function of NR2F1-AS1 in IVDD is scarcely researched. Therefore, it is critical to make certain the expression and effect of NR2F1-AS1 in IVDD.

Evidence is mounting that microRNAs (miRNAs) subserve the development of IVDD. By reviewing previous studies, we concluded that miRNAs’ expression is closely related to nucleus pulposus (NP) cell apoptosis and proliferation [16–20]. As a miRNA, miR-145-5p is confirmed to contribute to the progression of inflammatory diseases such as myocardial infarction[21], spinal cord injuries[22], and chronic glomerulonephritis[23]. Additionally, miR-145-5p aggravates rheumatoid arthritis (RA) by activating the NF-κB pathway and enhances the secretion of matrix metalloproteinase-9 (MMP-9)[24]. Disappointingly, the mechanism of miR-145-5p in IVDD remains largely unknown.

The forkhead box protein O (FOXO) family, also known as forkhead proteins, has four subtypes in mammals, namely FOXO1 (FKHR), FOXO3 (fkhrl1), FOXO4 (AFX) and FOXO6. FOXO1 plays a vital role in cell cycle control, apoptosis, metabolism and adipocyte differentiation[25]. Also, the FOXO1 transcription network is critical in regulating homeostasis and ECM, and it mediates the abnormal expression of these pathways observed in the pathogenesis of osteoarthritis (OA)[26]. Wang A et al. stated that MEG3 facilitates OA chondrocytes’ proliferation and hampers their apoptosis and ECM degradation through the miR-361-5p/FOXO1 pathway[27]. Nevertheless, the mechanism of FOXO1 in IVDD remains elusive.

This study seeks to characterize the function of NR2F1-AS1 in IVDD and to reveal its underlying mechanisms. Our findings imply that NR2F1-AS1 was upregulated in the intervertebral disc tissues from IVDD patients, whereas miR-145-5p was downregulated. NRF2F1-AS1 overexpression promoted IL-1β-mediated NP cell apoptosis, up-regulates ECM-related genes, and inhibited miR-145-5p level. The bioinformatic analysis showed that NR2F1-AS1 acts as a potential ceRNA on miR-145-5p, which targets FOXO1. Thus, we guessed NR2F1-AS1 influences IVDD progression via the miR-145-5p/FOXO1 axis. We hope this study provides novel mechanisms and potential options for IVDD treatment.

## 2. Methods and materials

### 2.1 Patients and sample collection

This research was granted by the Research Ethics Committee of the Zhongnan Hospital of Wuhan University Wuhan University (Approved number: WUZN-2019-0344). Human NP specimens were harvested from patients with idiopathic scoliosis (non-IVDD, n = 21) and patients with IVDD (n = 35) (see Table 2 for specific information). The patients were diagnosed as lumbar disc herniation or lumbar disc herniation combined with spinal stenosis and were treated by discectomy via a posterior. Those patients were excluded if they have bone metabolic disease, congenital bone malformation, gout, renal dysfunction, or hypercalcemia. Intervertebral disc tissues were collected during the surgery and immediately frozen in liquid nitrogen at −80°C. Immunohistochemistry (IHC) was performed for detecting Caspase3 (1:100, ab32351, Abcam, USA) in the intervertebral disc tissues [28]. IVDD patients with degenerative spinal stenosis, tumor, infection, or a prior lumbar disc surgery were excluded. All patients underwent routine preoperative lumbar MRI, and the degree of IVDD was analyzed with the modified Pfirrmann grade based on magnetic resonance imaging (MRI) T2 weighted images and the severity of degeneration. Pfirrmann grade I indicates a normal, healthy disc as only found in children, whereas Pfirrmann grade V indicates the most severe degree of degeneration. When it is above Pfirrmann grade III, the pore structure of bone endplate changes significantly and the number of pores decreases gradually [29–31].

**Table 1.** The primer sequences

Gene name	Forward primer	Reverse primer
NR2F1-AS1	5’-AACATCTGCTGCTGCAACCTGTG-3’	5’-AATGGCCACGCTGTATTGAC-3’
MiR-145-5p	5’-AACAAGGTCCAGTTTTCCCAGGA-3’	5’-CAGTGCAGGGTCCGAGGT-3’
TNF-α	5’-GGATTATGGCTCAGGGTCCA-3’	5’-ACATTCGAGGCTCCAGTGAA-3’
IL-1β	5’-GGCTCATCTGGGATCCTCTC-3’	5’-TCATCTTTTGGGGTCCGTCA-3’
Collagen II	5’-GCTCCCAGAACATCACCTACCA-3’	5’-ACAGTCTTGCCCCACTTACCG-3’
Aggrecan	5’-AGGTCGTGGTGAAAGGTGTTGTG-3’	5’-TGGTGGAAGCCATCCTCGTAG-3’
ADAMTS4	5’-GTCCTCCACACCCTAGCTTT-3’	5’-CAGGCAGGGAGAGACAAAGA-3’
MMP3	5’-AGTCTTCCAATCCTACTGTTGCT-3’	5’-TCCCCGTCACCTCCAATCC-3’
MMP13	5’-CTTCTTCTTGTTGAGCTGGACTC-3’	5’-CTGTGGAGGTCACTGTAGACT-3’
FOXO1	5’-GAGGAGCCTCGATGTGGATG-3’	5’-CCGAGATTTGGGGGAACGAA-3’
GAPDH	5’-GCTCTCTGCTCCTCCTGTTC-3’	5’-ACGACCAAATCCGTTGACTC-3’
U6	5’-CTCGCTTCGGCAGCACA-3’	5’-AACGCTTCACGAATTTGCGT-3’

**Table 2.** The clinical characteristics of IVDD patients and non-IVDD patients

Type	Sex	Age	Level	diagnosis(MRI)	Pfirrmann
non-IVDD 1	F	23	T3/T11	idiopathic scoliosis	I
non-IVDD 2	M	26	L2/L5	idiopathic scoliosis	I
non-IVDD 3	M	22	L1/L5	idiopathic scoliosis	II
non-IVDD 4	M	21	T12/L4	idiopathic scoliosis	I
non-IVDD 5	F	26	T6/T11	idiopathic scoliosis	II
non-IVDD 6	F	25	T3/T11	idiopathic scoliosis	I
non-IVDD 7	F	22	T8/L5	idiopathic scoliosis	I
non-IVDD 8	F	25	T6/L2	idiopathic scoliosis	II
non-IVDD 9	M	26	L1/L5	idiopathic scoliosis	I
non-IVDD 10	M	20	T12/L4	idiopathic scoliosis	II
non-IVDD 11	F	25	T6/T11	idiopathic scoliosis	II
non-IVDD 12	M	24	T3/T11	idiopathic scoliosis	II
non-IVDD 13	M	23	L2/L5	idiopathic scoliosis	I
non-IVDD 14	M	21	T5/T11	idiopathic scoliosis	I
non-IVDD 15	M	27	T5/T9	idiopathic scoliosis	I
non-IVDD 16	M	19	T5/T11	idiopathic scoliosis	II
non-IVDD 17	F	22	T2/T5	idiopathic scoliosis	I
non-IVDD 18	M	21	L1/L5	idiopathic scoliosis	I
non-IVDD 19	F	23	T2/T5	idiopathic scoliosis	I
non-IVDD 20	M	72	T6/T4	idiopathic scoliosis	II
non-IVDD 21	F	24	T12/L1	idiopathic scoliosis	II
IVDD 1	M	61	C4/C5, C5/C6, C6/C7	IVDD	III
IVDD 2	M	57	C5/C6	IVDD	II
IVDD 3	F	44	C5/C6	IVDD	II
IVDD 4	M	69	C5/C6, C6/C7	IVDD	IV
IVDD 5	M	70	C4/C5, C5/C6	IVDD	II
IVDD 6	F	52	C4/C5	IVDD	V
IVDD 7	M	69	C5/C6	IVDD	II
IVDD 8	F	72	C4/C5,C5/C6	IVDD	III
IVDD 9	F	56	C4/C5, C5/C6	IVDD	II
IVDD 10	F	72	C5/C6	IVDD	IV
IVDD 11	M	73	C5/C6	IVDD	V
IVDD 12	M	59	C6/C7	IVDD	II
IVDD 13	F	57	C4/C5, C5/C6, C6/C7	IVDD	IV
IVDD 14	M	45	C5/C6,C6/C7	IVDD	III
IVDD 15	F	61	C6/C7	IVDD	IV
IVDD 16	M	56	C5/C6, C6/C7	IVDD	V
IVDD 17	M	78	C5/C6	IVDD	II
IVDD 18	M	70	C6/C7	IVDD	III
IVDD 19	F	53	C6/C7	IVDD	II
IVDD 20	F	58	C4/C5, C5/C6, C6/C7	IVDD	IV
IVDD 21	F	46	C4/C5, C5/C6	IVDD	III
IVDD 22	F	51	C3/C4	IVDD	II
IVDD 23	M	42	C4/C5, C5/C6	IVDD	IV
IVDD 24	M	46	C5/C6	IVDD	III
IVDD 25	F	49	C5/C6	IVDD	V
IVDD 26	M	68	C4/C5, C5/C6, C6/C7	IVDD	III
IVDD 27	M	73	C5/C6, C6/C7	IVDD	III
IVDD 28	M	71	C6/C7	IVDD	IV
IVDD 29	M	59	C5/C6, C6/C7	IVDD	III
IVDD 30	M	67	C4/C5, C5/C6, C6/C7	IVDD	V
IVDD 31	F	62	C4/C5, C5/C6	IVDD	IV
IVDD 32	M	61	C5/C6	IVDD	IV
IVDD 33	F	63	C4/C5, C5/C6	IVDD	V
IVDD 34	M	72	C4/C5	IVDD	V
IVDD 35	F	74	C5/C6	IVDD	V

F:Female M:Male

### 2.2 Cell culture and treatment

Human denatured NP cells were extracted from NP tissues of IVDD patients. They were then resuspended in the RPMI-1640 complete culture medium comprising 10% fetal bovine serum and 1% penicillin/streptomycin and incubated at 37°C with saturated humidity and 5% CO_2_. The medium was substituted once every 2 to 3 days. When the cells were about to confluence, 0.25% trypsin was used for trypsinization and sub-culture. To establish an *in-vitro* cell model of IVDD, normal NP cells were processed with 20 ng/mL of interleukin (IL)-1β for 48 hours. Untreated NP cells were used as control cells [32].

### 2.3 Cell transfection

pcDNA empty vector (NC), pcDNA-lncRNA NR2F1-AS1 (lncRNA NR2F1-AS1), lncRNA NR2F1-AS1’s short hairpin RNA negative control (sh-NC), lncRNA NR2F1-AS1’s short hairpin RNA (sh-NR2F1-AS1), miRNA control (miR-NC), and miR-145-5p mimics were acquired from GenePharma Co., Ltd. (Shanghai, China). Human NP cells were seeded into 24-well plates (3 × 10^5^ cells/well) and cultured at 37°C with 5% CO_2_ for 24 hours before the transfection. The above vectors were transfected into NP cells with Lipofectamine®3000 (Invitrogen; Thermo Fisher Scientific, Inc.) as per the supplier’s specifications. The transfection validity was determined by quantitative reverse transcription-polymerase chain reaction (qRT-PCR). Finally, the cells were maintained at 37°C with 5% CO_2_ for 24 hours for later use [33].

### 2.4 Quantitative reverse transcription-polymerase chain reaction (qRT-PCR)

The total RNAs cells or tissues were separated with the TRIzol reagent (Invitrogen, Carlsbad, CA, USA) and the concentration was examined, the miRNA and mRNA were subjected to reverse transcription into cDNA using the One Step PrimeScript miRNA cDNA synthesis kit (Bao Biological Engineering Co., Ltd., Dalian, China) and PrimeScript RT kit (Invitrogen, Shanghai, China), respectively. We then implemented qRT-PCR by utilizing SYBR®Premix-Ex-Taq™ (Takara, TX, USA) and the ABI7300 system. The expression profiles of NR2F1-AS1, miR-145-5p and FOXO1 were assessed with the 2^−ΔΔCt^ method (U6 served as a housekeeping gene for miR-145-5p, and GAPDH was a housekeeping gene for NR2F1-AS1, FOXO1, MMP3, MMP13, ADAMTS4, aggrecan, and Collagen II). The primers were synthesized by Shanghai Sangon Biotech Co., Ltd. The primer sequences are exhibited inTable 1. qRT-PCR was conducted with 40 cycles of pre-denaturation at 95°C for 30 s, denaturation at 95°C for 5 s, and annealing/extension at 60°C for 30 s. The relative expression of the target gene was analyzed by the 2^−∆∆CT^ method. ∆CT = target gene – GAPDH, while ∆∆ = ∆CT experiment group -∆CT control group [34].

### 2.5 TdT-mediated dUTP nick end labeling (TUNEL)

Each group of cells in the plates were treated as described above. Then, the culture medium was discarded, and the cells were rinsed with PBS. Afterward, the cells were immobilized with immunostaining fixative solution for 30–60 minutes and flushed with PBS. Then, the immunostaining washing solution was added for ice incubation for 2 minutes. Next, 50 μL TUNEL detection solution was added to the sample and maintained for 60 minutes at 37°C in the dark. Subsequently, the cells were flushed 3 times with PBS. After mounting with the antifade mounting medium, the cells were reviewed under a fluorescence microscope. The excitation light was 450–500 nm, and the emission light was 515–565 nm (green fluorescence). Five fields of view were randomly chosen for each sample, and the apoptotic rate = apoptotic cells/total cells×100%[35].

### 2.6 Cellular immunofluorescence

miR-NC and miR-145-5p were transfected into NP cells according to the instructions. Then, the IL-1β-treated cells were seeded on 24-well plates, and the coverslip was prepared. After 48 hours, the cells growing on the coverslip were cleaned 3 times with PBS, secured with 4% paraformaldehyde for 30 minutes, and permeated with 0.1% Triton X-100 for 10 minutes. After the cells were rinsed 3 times with PBS, they were kept with 3% H_2_O_2_ for 10 minutes and blocked with 10% goat serum + 3% bovine serum albumin for 30 minutes. Afterward, they were maintained with the primary antibody of p-FOXO1 (Proteintech, USA; 1:100) at 4°C overnight and goat anti-rabbit IgG (H + L) (1:500) for 1 hour at room temperature (RT). The nucleus was dyed with DAPI, and the cells were viewed after mounting and photographing [36].

### 2.7 Annexin V-FITC-PI apoptosis detection assay

Annexin V-FITC-PI apoptosis detection kit (Cat:40,302, Yeasen, Shanghai, China) was used for evaluating apoptosis. NP cells were trypsinized with 0.25% trypsin, then collected by centrifugation (1000 rpm, 5 min). PBS was used for washing the cells three times, and the cells were incubated with containing 200 μL Annexin V-FITC and maintained in the dark for 10 minutes. They were then flushed with 200 μL PBS, and 10 μL PI was added. Cell apoptosis was examined by flow cytometry (FCM) (Beckman Coulter) [37].

### 2.8 RNA fluorescence in situ hybridization (FISH)

NP cells were grown on 4-chamber glass slides for 48 hours. After rinsing with PBS, the cells were immobilized with 3.7% paraformaldehyde, permeated with 70% ethanol, and then rehydrated in 2× SSC and 50% formamide for 5 minutes. The cells were hybridized with biotin-labeled NR2F1-AS1 and miR-145-5p probe mixture overnight at 42°C. The mixture contained 10% dextran sulfate, 5× Denhardt reagent, 2× SSC, 50% formamide and 100 μg/mL denatured and fragmented salmon sperm DNA. Nonspecific probes were removed by 0.5× SSC comprising 50% formamide at 37°C. The anti-biotin monoclonal antibody and the secondary antibody conjugated to AlexaFluor®647 were utilized to detect biotin-labeled NR2F1-AS1 and miR-145-5p. The cells were cleaned with PBS and then put on the coverslip with a reagent containing DAPI (Cell Signaling Technology, Boston, MA, USA) [38].

### 2.9 Protein isolation and Western blot (WB)

After the cells were processed with varying factors, the primary culture medium was discarded. The RIPA (containing 1% PMSF) lysis buffer was employed to lyse the cells, which were collected through low-speed centrifugation to isolate the total protein. Then, the protein quantification was made with the Bradford method, and the samples were boiled for 5 minutes and centrifuged for 30 s after ice-cooling. Afterward, the supernatant was taken for polyacrylamide gel electrophoresis, and 30 μg of total protein was loaded onto a 10% SDS-PAGE gel and transferred to PVDF membranes (Millipore, USA). After being sealed with 10% skim milk powder solution for two hours, the membranes were maintained with the primary antibodies of FOXO1 (Abcam, 1:1000, ab52857, MA, USA), p-FOXO1 (1:1000, ab259337), Bax (1:1000, ab32503), Bcl2 (1:1000, ab182858), and GAPDH (ab9485, 1:1000) overnight. After that, the membranes were flushed with TBST twice and kept with the fluorescein-labeled secondary antibody at RT for 1 hour. Finally, the membranes were flushed 3 times, exposed with the ECL chromogenic agent, and imaged with the membrane scanner [39].

### 2.10 Dual-luciferase reporter assay

TargetScan software indicated that FOXO1 was an underlying target of miR-145-5p, while miR-145-5p was that of NR2F1-AS1. The reporter plasmids of wild-type and mutant NR2F1-AS1 and SAMD3-3’UTR were constructed, and miR-145-5p mimics and their negative controls were transfected into NP cells. The experiment was carried out 48 hours later as per the dual-luciferase reporter assay instructions (Promega, Madison, WI, USA). The relative fluorescence intensity of different treatment groups was estimated following the ratio of firefly fluorescence intensity/renilla fluorescence intensity detected by the microplate reader [40].

### 2.11 RNA immunoprecipitation (RIP)

RIP was conducted with the Magna RIP Kit (EMD Millipore, Billerica, MA, United States) as per the manufacturer’s instructions. Following cell lysis with the RIP lysis buffer, the human anti-Ago-2 antibody (microporous) or the control antibody (normal mouse immunoglobulin, micropores) was added and maintained overnight at 4°C. The expression of NR2F1-AS1 and FOXO1 was assessed by qRT-PCR [41].

### 2.12 Statistical analysis

The SPSS17.0 statistical software (SPSS Inc., Chicago, IL, USA) was adopted for analysis. Measurements were presented as mean ± standard deviation (x ± s). Pearson analysis was adopted to determine the correlation between NR2F1-AS1 and miR-145-5p in NP tissues. StarBase (https://starbase.sysu.edu.cn/) was utilized to predict the miRNA targets of lncRNAs [42]. The multi-factor comparison was made by one-way analysis of variance, and *t* test was utilized for comparison between the two groups. *P*< 0.05 signified statistical significance.

## 3. Results

### 3.1 LncRNA NR2F1-AS1 was up-regulated, and miR-145-5p was down-regulated in NP tissues of IVDD patients

**Figure 1.**
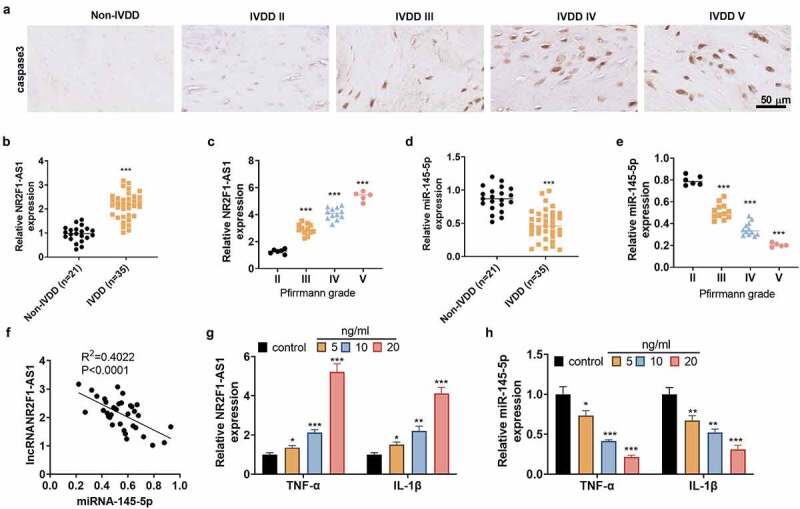
NR2F1-AS1 was up-regulated, and miR-145-5p was down-regulated in NP tissues of IVDD patients.

To figure out the expression of LncRNA NR2F1-AS1 in NP cells, we harvested human intervertebral disc tissues from IVDD patients and non-IVDD patients. IHC was performed for detecting Caspase3 in the tissues. We found that intervertebral disc tissues from non-IVDD patients had low expression of Caspase3, whereas Caspase3 was gradually increased with the increasing of Pfirrmann grades (Figure 1a). The NR2F1-AS1 expression was detected by RT-PCR. NR2F1-AS1 was confirmed to be up-regulated in intervertebral disc tissues from IVDD patients compared with that in the non-IVDD patients (Figure 1b). The expression level of NR2F1-AS1 was strengthened with the deterioration of IVDD (Figure 1c). The miR-145-5p expression was examined by qRT-PCR, and it was confirmed to be down-regulated in intervertebral disc tissues from IVDD patients compared with that in the non-IVDD patients (Figure 1d) and gradually decreased with the deterioration of IVDD (Figure 1e). Linear regression analysis showed that NR2F1-AS1 was reversely related to miR-145-5p (figure 1f). To probe the correlation between inflammation and the expression of NR2F1-AS1 and miR-145-5p, we treated normal NP cells with TNF-α (5, 10, 20 ng/mL) and IL-1β (5,10, 20 ng/mL) for 24 hours. As a result, TNF-α or IL-1β dose-dependently induced up-regulation of NR2F1-AS1 and down-regulation of miR-145-5p in NP cells versus the Con group (Figure 1g, h). These outcomes disclosed that NR2F1-AS1 and miR-145-5p were abnormally expressed in IVDD and contributed to its pathogenesis.

A: IHC was performed for detecting Caspase3 in the intervertebral disc tissues from IVDD or non-IVDD patients. Scale bar = 50 μm. B. The NR2F1-AS1 expression in intervertebral disc tissues from IVDD or non-IVDD patients was compared by qRT-PCR. C: The lncRNA NR2F1-AS1 level in intervertebral disc tissues IVDD patients with different Pfirmann grades. D: The miR-145-5p level in intervertebral disc tissues from IVDD or non-IVDD patients was determined by qRT-PCR. E: The miR-145-5p level gradually decreased with the deterioration of IVDD. F: Pearson analysis showed that NR2F1-AS1 was reversely related to miR-145-5p. R^2^ = 0.4022, *P*< 0.0001. *P < 0.05, ** *P*< 0.01, *** *P*< 0.001 (vs. Non-IVDD or II group). G-H. NP cells were dealt with TNF-α (5–20 ng/ml) or IL-1β (5–20 ng/ml) for 48 hours. *P < 0.05, ** *P*< 0.01, *** *P*< 0.001 (vs. control group). N = 3.

### 3.2 NR2F1-AS1 facilitated extracellular matrix denaturation and apoptosis in human NP cells

**Figure 2.**
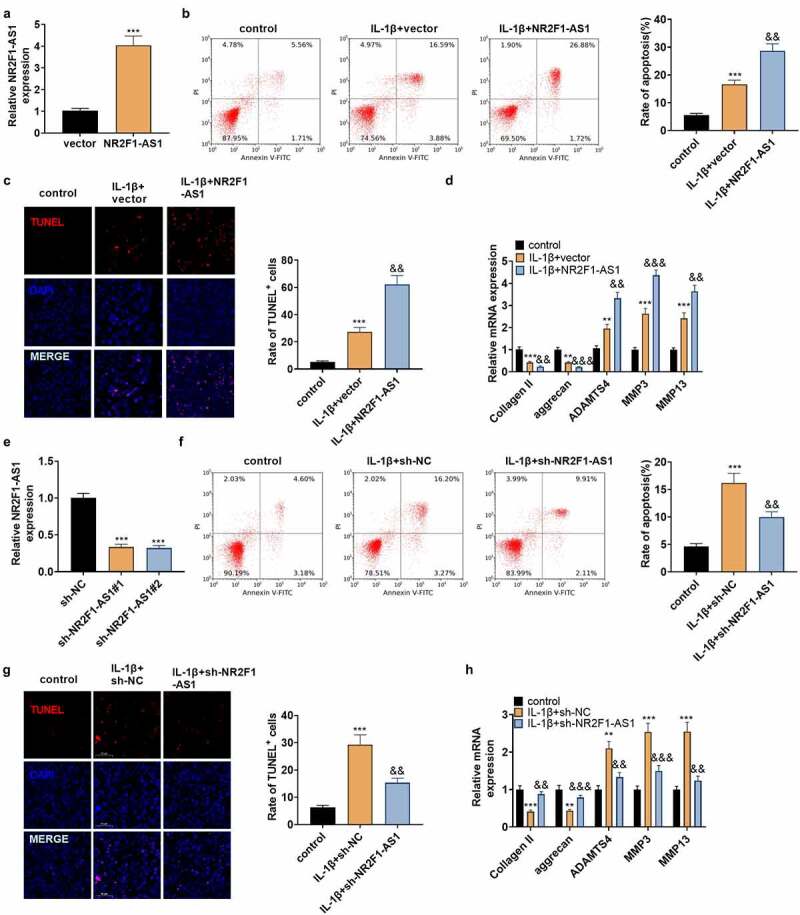
NR2F1-AS1 enhanced ECM degeneration and human NP cell apoptosis.

To make certain the impact of NR2F1-AS1 on NP cells, NP cells were separated from NP tissues of IVDD patients and transfected with NR2F1-AS1 overexpression plasmids. qPT-PCR verified that NR2F1-AS1 was up-regulated in NP cells (Figure 2a). IL-1β was adopted to treat the transfected NP cells, and NP cell apoptosis was verified by FCM and TUNEL staining. The outcomes illustrated that by contrast with the control group, the apoptotic rate and TUNEL-positive cell number in IL-1β-treated NP cells increased significantly, and the increase was strengthened after NR2F1-AS1 overexpression (Figure 2b, c). The profiles of Collagen II, aggrecan, ADAMTS4, MMP3, and MMP13 were examined by qRT-PCR. Notably, compared with the control group, ADAMTS4, MMP3, and MMP13 were highly expressed, while collagen II and aggrecan were down-regulated in IL-1β-treated NP cells. After NR2F1-AS1 overexpression, Collagen II, and aggrecan was downregulated, and ADAMTS4, MMP3, and MMP13 were further upregulated (compared with IL-1β+vector group, Figure 2d). Additionally, we transfected IL-1β-treated NP cells with sh-NR2F1-AS1 to reversely verify the influence of NR2F1-AS1 on NP cells. As testified by qPT-PCR, NR2F1-AS1 was down-regulated in NP cells (vs. IL-1β+sh-NC group, Figure 2e). NP cell apoptosis was lower in the IL-1β+NR2F1-AS1 group than that of the IL-1β+sh-NC group (Figure 2f, g). Followed by NR2F1-AS1 downregulation, the expression of ADAMTS4, MMP3, and MMP13 was decreased, while collagen II and aggrecan were up-regulated (p < 0.05 vs.the IL-1β+sh-NC group, Figure 2h). Thus, NR2F1-AS1 intensifixed apoptosis and ECM degeneration in NP cells, while inhibiting NR2F1-AS1 had the reverse effect.

NR2F1-AS1 overexpression plasmids were transfected into the denatured human NP cells dealt with IL-1β (20 ng/ml) for 48 hours. A: The NR2F1-AS1 expression was evaluated by qRT-PCR. B: FCM gauged the apoptotic rate of human NP cells. C: TUNEL assessed the number of TUNEL-positive cells. D: qRT-PCR monitored the expression of collagen II, aggrecan, ADAMTS4, MMP3, and MMP13 in human NP cells.The sh-LncRNA NR2F1-AS1 was transfected into denatured human NP cells. A: qRT-PCR examined the level of NR2F1-AS1. B: The apoptotic rate of human NP cells was measured by FCM. C: TUNEL checked the number of TUNEL-positive cells in human NP cells. D: The expression of collagen II, aggrecan, ADAMTS4, MMP3, and MMP13 in human NP cells was determined by qRT-PCR. ** *P*< 0.01, ***P< 0.001 (vs. control group), && *P*< 0.01, &&& *P*< 0.001 (vs.IL-1β+vector or sh-NC). (N = 3, one-way ANOVA followed by Tukey post hoc test).

### 3.3 miR-145-5p hindered extracellular matrix degeneration and apoptosis in human NP cells

**Figure 3.**
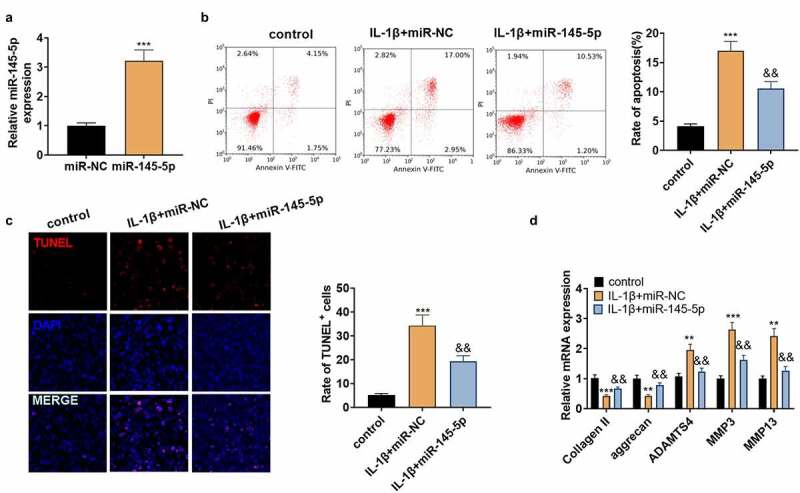
miR-145-5p dampened ECM degeneration and NP cell apoptosis.

The above study revealed that miR-145-5p was down-regulated in NP tissues of IVDD patients, but it is not clear about the role of miR-145-5p in IVDD. Hence, NP cells were transfected with miR-145-5p mimics, and then qRT-PCR revealed that 145–5p was up-regulated in NP cells (vs.IL-1β+miR-NC group, Figure 3a). Meanwhile, in comparison to the IL-1β+miR-NC group, up-regulating miR-145-5p reduced the apoptotic rate of NP cells and the TUNEL-positive cell number (Figure 3b, c). Subsequently, qRT-PCR manifested that ADAMTS4, MMP3, and MMP13 were significantly down-regulated following the transfection of miR-145-5p mimics, but the expression of collagen II and aggrecan were increased (Figure 3d). These results manifested that up-regulating miR-145-5p repressed NP cell apoptosis and ECM degradation.

We transfected the miR-145-5p mimics into NP cells dealt with IL-1β (20 ng/ml) for 48 hours. A: The miR-145-5p expression was verified by qRT-PCR. B: The apoptotic rate of human NP cells was monitored by FCM. C: TUNEL tested the number of TUNEL-positive cells in human NP cells. D: qRT-PCR monitored the expression of collagen II, aggrecan, ADAMTS4, MMP3, and MMP13 in human NP cells. ** *P* < 0.01, *** *P* < 0.001 (vs. control group). && *P*< 0.01 (vs. IL-1β+miR-NC group). (N = 3, one-way ANOVA followed by Tukey post hoc test).

### 3.4 NR2F1-AS1 targeted miR-145-5p

**Figure 4.**
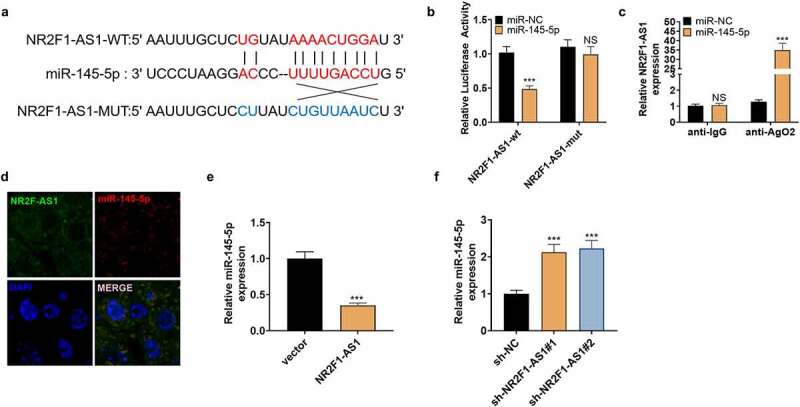
NR2F1-AS1 targeted miR-145-5p.

We searched the target genes of NR2F1-AS1 on StarBase (http://starbase.sysu.edu.cn/index.php), which revealed that miR-145-5p was targeted by NR2F1-AS1 (Figure 4 A). The dual-luciferase reporter assay was performed to affirm the association between the two. Notably, miR-145-5p overexpression weakened the luciferase activity of NP cells transfected with NR2F1-AS1-WT, while it had little impact on that of NP cells transfected with the NR2F1-AS1-mut vector (Figure 4b). Furthermore, the RIP experiment was implemented to clarify the association between the two. It turned out that after the miR-145-5p transfection, the amount of NR2F1-AS1 precipitated in the Ago2 antibody group was more than that in the IgG group, hinting that NR2F1-AS1 bound to Ago2 via miR-145-5p (Figure 4c). The FISH experiment exhibited that NR2F1-AS1 and miR-145-5p were expressed in the cytoplasm (Figure 4d). Finally, we observed that overexpressing NR2F1-AS1 reduced the miR-145-5p expression, while knocking down NR2F1-AS1 exerted opposite effects (Figure 4e-f). The above results verified that NR2F1-AS1 targeted miR-145-5p.

A: The binding site between NR2F1-AS1 and miR-145-5p was analyzed by the Starbase database. B: Dual-luciferase reporter assay illustrated that miR-145-5p enhanced the luciferase activity of NR2F1-AS1-WT. C: RIP showed that NR2F1-AS1 bound to miR-145-5p. D: FISH manifested that NR2F1-AS1 co-localized with miR-145-5p in the cytoplasm. E and F: Overexpressing NR2F1-AS1 reduced miR-145-5p expression, while inhibiting NR2F1-AS1 had the opposite effect. NS*P*>0.05, ***P< 0.001 (vs. miR-NC) (N = 3, by Student’s t-test or one-way ANOVA followed by Tukey post hoc test).

### 3.5 NR2F1-AS1 strengthened extracellular matrix degradation and apoptosis in human NP cells through miR-145-5p/FOXO1

**Figure 5.**
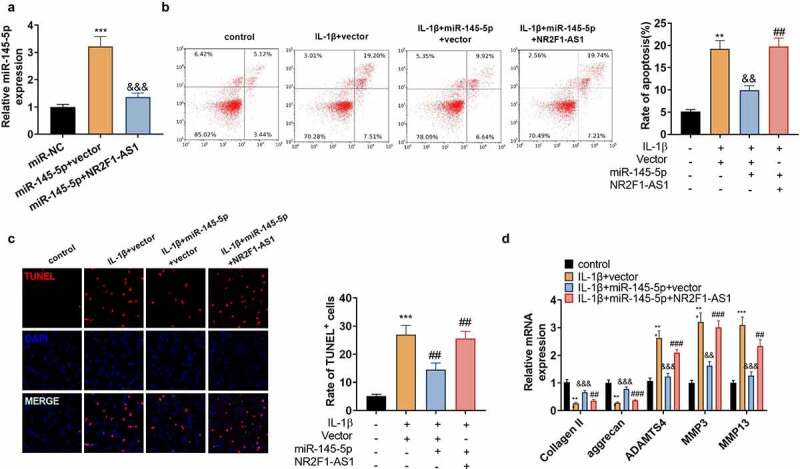
NR2F1-AS1 strengthened the ECM denaturation and apoptosis in human NP cells via miR-145-5p/FOXO1.

The above data corroborated that NR2F1-AS1 targeted miR-145-5p. Next, we probed whether NR2F1-AS1 exerted a pro-apoptotic effect on NP cells via miR-145-5p. We transfected NP cells with miR-145-5p mimics with, or without NR2F1-AS1 overexpression plasmids to determine whether NR2F1-AS1 enhanced NP cell apoptosis and ECM degradation by targeting miR-145-5p. qRT-PCR data illustrated that compared with the miR-145-5p+vector group, miR-145-5p was down-regulated after overexpressing NR2F1-AS1 (Figure 5a). Besides, NR2F1-AS1 overexpression led to strengthened NP cell apoptosis versus the IL+1β+miR-145-5p+vector group (Figure 5b, c). qRT-PCR confirmed that ADAMTS4, MMP3, and MMP13 were up-regulated, while collagen II and aggrecan were down-regulated following NR2F1-AS1 overexpression versus the IL+1β+miR-145-5p+vector group (Figure 5d). These outcomes uncovered that NR2F1-AS1 increased ECM degradation and apoptosis in human NP cells by down-regulating miR-145-5p.

The miR-145-5p mimics and, or NR2F1-AS1 overexpression plasmids were transfected into NP cells dealt with IL-1β (20 ng/ml) for 48 hours. A: qRT-PCR monitored the miR-145-5p expression. B: FCM was adopted to verify the apoptotic rate of human NP cells. C: TUNEL estimated the number of TUNEL-positive cells in human NP cells. D: The profiles of collagen II, aggrecan, ADAMTS4, MMP3, and MMP13 in human NP cells were monitored by qRT-PCR. *** *P*< 0.001 (vs. control group), &&*P*< 0.01, &&&*P*< 0.001 (vs. IL-1β+vector group), ##*P*< 0.01, ###*P*< 0.001 (vs. IL-1β+miR-NC+vector group) (N = 3, one-way ANOVA followed by Tukey post hoc test).

### 3.6 miR-145-5p targeted FOXO1

**Figure 6.**
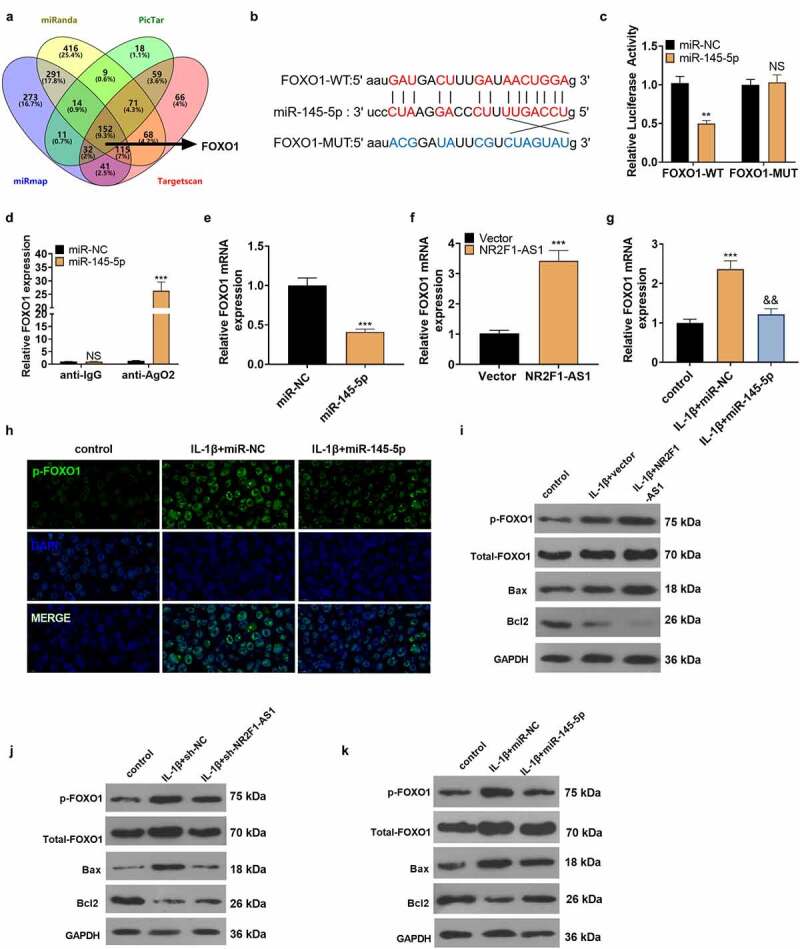
miR-145-5p targeted FOXO1.

**Figure 7.**
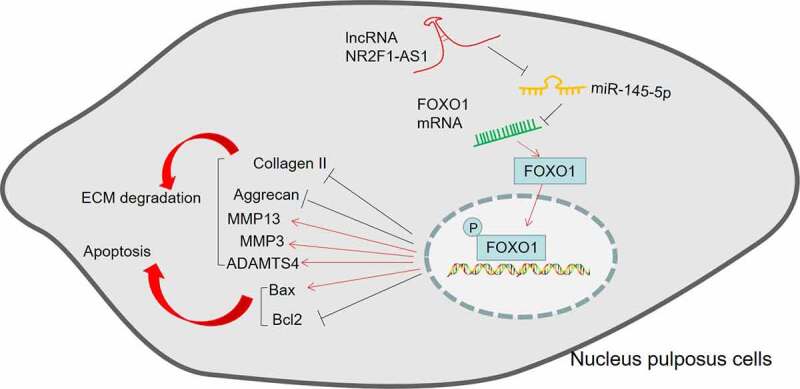
The mechanistic diagram.

By searching the miRanda, PicTar, miRmap, and Targetscan websites, we discovered that miR-145-5p has a total of 152 potential mRNA targets, including FOXO1 (Figure 6a). Starbase software revealed that miR-145-5p had binding sites with FOXO1 (Figure 6b). To make certain whether miR-145-5p is bound to the predicted target site in FOXO1, we constructed wild-type and mutant (it was assumed that the binding site of miR-145-5p was mutated) FOXO1 luciferase reporter vectors. As expected, miR-145-5p overexpression distinctly abated the luciferase activity of NP cells transfected with FOXO1-WT, but it had no influence on the that of FOXO1-MUT (Figure 6c). Additionally, RIP analysis signified that FOXO1 and miR-145-5p were rich in Ago2 microribonucleoprotein complexes, indicating that Ago2 is directly bound to FOXO1 and miR-145-5p in NP cells (Figure 6d). miR-145-5p mimics were transfected into NP cells, and qRT-PCR confirmed that the FOXO1 mRNA expression was suppressed versus the control group (Figure 6e). In addition, we conducted qRT-PCR, which displayed that up-regulation of NR2F1-AS1 uplifted the mRNA expression of FOXO1 versus the Vector group (figure 6f). Then miR-145-5p mimics were transfected into IL-1β-processed NP cells. As testified by qRT-PCR, FOXO1 was notably up-regulated in IL-1β-induced NP cells versus the Con group, and up-regulation of miR-145-5p reduced FOXO1 expression in NP cells (Figure 6g). Cellular immunofluorescence showed that the fluorescence intensity of p-FOXO1 in NP cells transfected with miR-145-5p was weaker than that of the IL-1β+miR-NC group (Figure 6h). IL-1β-treated NP cells were transfected with NR2F1-AS1, sh-NR2F1-AS1, and miR-145-5p. WB results illustrated that by contrast with the IL-1β+vector group, NR2F1-AS1 increased p-FOXO1, total FOXO1 and the expression of the pro-apoptotic protein Bax, and repressed the expression of the anti-apoptotic protein Bcl2. On the contrary, down-regulating NR2F1-AS1 or up-regulating miR-145-5p exerted the opposite effects (Figure 6 I-K). The above results verified that FOXO1 was the target of miR-145-5p. miR-145-5p was negatively correlated with FOXO1, and NR2F1-AS1 was positively correlated with FOXO1 (Figure 7).

A: By searching the miRanda, PicTar, miRmap, and Targetscan websites, we discovered that miR-145-5p had 152 binding sites, including FOXO1. B: Bioinformatics analysis predicted the binding site between miR-145-5p and FOXO1. C: The dual-luciferase report assay testified that miR-145-5p facilitated the luciferase activity of FOXO1-WT. D: RIP showed that FOXO1 is bound to miR-145-5p. NS*P*>0.05, **P< 0.01, ***P< 0.001 (vs. miR-NC). E: After up-regulating miR-145-5p, the FOXO1 profile decreased. F: qRT-PCR tested FOXO1 expression after overexpressing NR2F1-AS1. G: IL-1β treatment up-regulated FOXO1 in NP cells, and up-regulating miR-145-5p abated the FOXO1 expression. H: Cellular immunofluorescence detection revealed that the fluorescence intensity of p-FOXO1 was weakened after up-regulating miR-145-5p. I-K: WB detected the phosphorylation of FOXO1 and the expression of Bax and Bcl2 after up-regulating NR2F1-AS1, down-regulating NR2F1-AS1, and overexpressing miR-145-5p in IL-1β-treated NP cells. ***P< 0.001, &&*P*< 0.01 (vs.IL-1β+miR-NC) (N = 3, by Student’s t-test).

NR2F1-AS1 was overexpressed during IVDD progression. Tthe lncRNA NR2F1-AS1/miR-145-5p/FOXO1 axis in regulating the apoptosis and ECM degradation.

## 4. Discussion

IVDD is one primary reason for backache, which is a serious socio-economic burden. IVDD is usually defined by changes in tissue function and structure, including excessive ECM degradation and increased intervertebral disc cell senescence and death [43]. Inflammatory processes exacerbated by TNF-α, IL-1, and IL-6 are key mediators of IVDD and backache [44]. Therefore, the association between lncRNAs, ECM degradation and inflammation was studied in this paper. The possible mechanism was that NR2F1-AS1 increases the FOXO1 expression by down-regulating miR-145-5p, thus facilitating inflammation-mediated NP cell apoptosis and ECM degradation (Figure 7).

Emerging evidence shows that noncoding RNAs exert an essential role in the biological process of IVDD [5,6]. For example, the ectopic expression of LINC00958 promotes NP cell proliferation, dampens the expression of collagen II and aggrecan, and facilitates the expression of MMP-2 and MMP-13[45]. Overexpressing lncRNA RMRP enhances NP cell growth, elevates the expression of collagen II and aggrecan, and abates the expression of MMP13 and ADAMTS4[46]. Besides, MALAT1[47], lncRNA TRPC7-AS1[48], and lncRNA-RP11-296A18.3 [49] all contribute to IVDD. The role of lncRNA NR2F1-AS1 in tumors has been extensively studied [50,51]. Here, we confirmed that NR2F1-AS1 was up-regulated during IVDD progression. NR2F1-AS1 overexpression increased IL-1β-mediated NP cell apoptosis and ECM degradation, suggesting NR2F1-AS1 is a potential biomarker in IVDD evolvement and treatment.

miRNAs have been confirmed to contribute to diversified pathological processes of IVDD, such as apoptosis, ECM degradation, cell proliferation and inflammation[52]. For instance, Zhao K et al. found that miRNA-143 enhances NP cell apoptosis by directly targeting BCL2, providing an underlying treatment option for IVDD[53]. Wang J et al. stated that the miR-154 level is elevated in NP cells in IVDD patients. Besides, the inhibition of miR-154 strengthens the protein profile of collagen II and aggrecan and reduces the mRNA expression of MMP13 and ADAMTS4, while miR-154 overexpression reverses the effect[54]. Also, Zhang et al. confirmed that miR-222 is uplifted in IVDD tissues and LPS-treated nucleus pulposus cells, and miR-222 significantly promotes the generation of TNF-α, IL-1β, and IL-6[55]. Moreover, miR-98, miR-149, miR-27b, and miR-133a are all related to the degree of IVDD [56–59]. Nevertheless, the function of miR-145-5p in IVDD remains elusive. Here, we testified that miR-145-5p was downregulated in IVDD patients and overexpressing miR-145-5p relieved IL-1β induced NP cell apoptosis and ECM degradation. As a downstream target of NR2F1-AS1, miR-145-5p was inhibited by the latter. The rescue experiments indicated that the NR2F1-AS1/ miR-145-5p has a potential role during IVDD progression.

A previous report indicates that FOXOs are key regulators of IVDD homeostasis during aging. Maintaining or restoring FOXO expression can be used as a therapeutic strategy to delay the onset of IVDD [60,61]. FOXO3, one member of FOXO families, has potent effects in protecting nucleus pulposus cells against apoptosis by repressing inflammation, ECM degradation, oxidative stress. [62–64] In addition, studies by Chai X et al. showed that FOXO1 is up-regulated in LPS-treated NP cells, and overexpressing FOXO1 aggravates LPS-induced NP cell damages [65]. Previous studies have found that FOXO1, a vital transcription factor in cells, promotes inflammatory reactions and oxidative stress [66,67]. FOXO1a also gets involved in IVDD progression by driving annulus fibrosus (AF) cells apoptosis through mitochondrial-related pathway[68]. which is consistent with our study. Here, we substantiated that FOXO1 was up-regulated in degenerated human NP cells, and miR-145-5p mimics could down-regulate FOXO1. At the same time, the expression of NR2F1-AS1 and FOXO1 was positively correlated in NP cells, suggesting that NR2F1-AS1 and miRNA-145-5p targeted and regulated FOXO1 to affect IVDD progression.

## 5. Conclusion

Collectively, this study demonstrated that NR2F1-AS1 modulates the FOXO1 axis by sponging miR-145-5p as a ceRNA, thus regulating NP cell apoptosis and ECM degradation. This research reveals the mechanism of the lncRNA NR2F1-AS1/miR-145-5p/FOXO1 axis in NP cell damage in in-vitro experiments, providing potential therapeutic targets for IVDD. However, further experiment should be conducted for confirming the role of lncRNA NR2F1-AS1/miR-145-5p/FOXO1 axis in IVDD animal model.

## Data Availability

The data sets used and analyzed during the current study are available from the corresponding author on reasonable request.

## Abbreviations

ceRNA: competitive endogenous RNA; ECM: extracellular matrix; FCM: Flow cytometry; FISH: fluorescence in situ hybridization; FOXO1: forkhead box protein O 1; IHC: immunohistochemistry; IL-1β: interleukin–1β; IVDD: Intervertebral disc degeneration; lncRNAs: long noncoding RNAs; LPS: lipopolysaccharide; MMP9: matrix metalloproteinase-9; MUT: mutant; NC: negative control; NP: nucleus pulposus; NR2F1-AS1: nuclear receptor subfamily 2 group F member 1 antisense RNA 1; NS: no significance; OA: osteoarthritis; OS: osteosarcoma; PVDF: polyvinylidene fluoride; RA rheumatoid arthritis; RIP: RNA immunoprecipitation; RT-PCR: Reverse transcription-polymerase chain reaction; shRNA: short hairpin RNA; TUNEL: TdT-mediated dUTP nick end labeling; UTR: untranslated region; WT: wild type.
